# Circulating tumor cells as a response monitor in stage IV non-small cell lung cancer

**DOI:** 10.1186/s12967-019-2035-8

**Published:** 2019-08-28

**Authors:** Stephanie N. Shishido, Anders Carlsson, Jorge Nieva, Kelly Bethel, James B. Hicks, Lyudmila Bazhenova, Peter Kuhn

**Affiliations:** 1 Michelson Center for Convergent Bioscience, Dornsife College of Letters, Arts and Sciences, University of Southern California, 1002 W Childs Way, MCB351, MC:3502, Los Angeles, CA 90089-3502 USA; 2 University of Southern California, 1441 Eastlake Avenue, Suite 3440, Los Angeles, CA 90033 USA; 3 Scripps Clinic, Department of Pathology, 10666 North Torrey Pines Road, MC211C, La Jolla, CA 92037 USA; 4 Moores Cancer Center, University of California San Diego, 3855 Health Sciences Dr, La Jolla, CA 92093 USA

**Keywords:** Non-small cell lung cancer, Circulating tumor cells, HD-SCA, Liquid biopsy

## Abstract

**Background:**

Monitoring circulating tumor cells (CTC) has been shown to be prognostic in most solid malignancies. There is no CTC assay in clinical use for lung cancer therapy monitoring due to inconclusive clinical utility data. Limited data has been published outside of the standard CTC enumerations, regarding clinical significance of phenotypic heterogeneity of CTCs in late stage NSCLC and its ability to correlate with treatment outcomes.

**Methods:**

In 81 patients with stage IV NSCLC, multiple timepoints for CTC analysis were collected after initiation of treatment across 139 lines of therapy using single cell high definition diagnostic pathology imaging of all nucleated cells from 362 peripheral blood samples as a liquid biopsy.

**Results:**

We analyzed the subset of 25 patients with complete time series data, totaling 117 blood samples, to determine the significance of HD-CTC kinetics during the initiation of treatment. These kinetics follow three distinct patterns: an increase in HD-CTCs with therapy (mean + 118.40 HD-CTCs/mL), unchanged HD-CTCs numbers (stable; mean 0.54 HD-CTCs/mL), and a decrease in HD-CTCs numbers (mean − 81.40 HD-CTCs/mL). Patients with an increasing CTC count during the first 3 months post initiation of new treatment had a better PFS and OS compared to the other groups. There was weak correlation between the absolute number of HD-CTCs at a single time point of therapy and patient outcomes (OS *p* value = 0.0754). In the whole cohort of 81 patients, HD-CTCs were detected in 51 (63%) patients at initiation of therapy with a median of 2.20 (range 0–509.20) and a mean of 26.21 HD-CTCs/mL (± 15.64).

**Conclusions:**

CTCs are identifiable in most patients with stage IV NSCLC. While absolute HD-CTC counts do not correlate with prognosis, the changes in CTC counts were predictive of survival in patients with metastatic lung cancer receiving chemotherapy. The level and dynamics of CTCs indicate very different biological and pharmacological phenomena at different stages of disease and timepoints of treatment, highlighting the complex role of CTCs in cancer research and clinical management.

## Background

Dissemination of tumor cells through the circulation may be key in the progression of solid tumors, including lung cancer. The management of NSCLC has shifted over the past decade. Molecular based treatment decisions are now considered standard of care, and improved outcomes are seen with targeted therapy compared to chemotherapy in certain subgroups. However, drug development faces several hurdles, including the lack of access to pharmacodynamic markers and longitudinally collected tumor specimens [1, 2]. Surprisingly, circulating tumor cell (CTC) isolation technologies have yet to perform robustly in NSCLC relative to the success of studies in small-cell lung cancer (SCLC) or prostate cancer. The use of liquid biopsies to assess CTC enumeration either as a whole, or for specific subgroups, could enhance the understanding of disease progression, and has already been shown to carry prognostic information in several cancer types, including lung cancer [3–8].

CTCs are defined as cells that have been released into the peripheral blood from either the primary tumor or metastatic sites. CTCs have been shown to fluctuate with therapy and predict response to certain agents, indicating the potential for these cells to serve as a predictive biomarker. It is important to note that molecular characterization and enumeration of CTCs has been conducted on several different cancer types using a variety of detection methods integrated with molecular characterization [9–11].

However, clinicians do not commonly use CTC information to make treatment decisions, despite its potential prognostic and predictive value [12, 13]. The scant amount of data regarding treatment and outcomes associated with CTC monitoring is surprising. One reason CTC monitoring has not been adopted in clinical lung cancer practice is the low prevalence or inability to detect CTCs in certain cancers, such as NSCLC [5–8]. As well as, there is a general lack of sufficient evidence for clinical utility in a specific context of use that can support clinical decision making to improved outcomes for NSCLC patients. Currently there are no FDA approved assays for the detection of CTCs in lung cancer patients. CellSearch^®^ (Veridex, Raritan NJ), the current clinical standard for CTC enumeration in breast, prostate and colorectal cancer, uses immunomagnetic enrichment of epithelial cell adhesion molecule (EpCAM) expressing cells, but is not approved in NSCLC. This may be due to the highly variable CTC detection rates that have been reported in stage IV NSCLC patients using CellSearch^®^, as well as the fact that tumor cells with low EpCAM expression such as NSCLC may escape detection by this method [6, 8, 14–21]. Furthermore, potential utility of CTCs in the clinical setting is limited by the several different methods and cellular thresholds that have been employed in CTC detection studies from a variety of NSCLC cohorts, restricting the possibility of comparative findings.

Here we use a non-enrichment based high-definition single cell assay (HD-SCA) workflow to detect CTCs based on morphology and high-throughput analysis in stage IV NSCLC patients. Assessment of CTCs using the HD-SCA workflow has previously been investigated in 78 early and late stage chemotherapy naïve lung cancer patients, which showed a high prevalence (61% positive) of CTCs in stage IV patients [22]. CTC positivity based on the HD-SCA workflow is inclusive of cellular morphology with interrogation of all nucleated cells from the liquid biopsy, enabling the possibility to serve as a disease and therapy resistance monitoring test, especially for patients with EpCAM low or negative disease. This study reports the analysis of CTCs with longitudinal surveillance of stage IV NSCLC patients entering different lines of therapy, in association with progression-free (PFS) and overall survival (OS).

## Methods

### Study design

This was a single institution prospective study of 113 patients with stage IV (AJCC 7.0) NSCLC with initial sample collection beginning between April 2009 and October 2010. Eligible patients had progressive disease starting a new line of therapy. The study was approved by the Institutional Review Board at the University of California San Diego. After informed consent was obtained peripheral blood samples were collected at the time of initiation of new therapy (time 0), 3 weeks, 3 months, and every 3 months thereafter until progression. Upon progression the collection schedule was restarted. Blood collection was stopped after 2 years, but patients were followed for survival. All patients had pathologically confirmed NSCLC. All patients had evaluable disease. Measurable disease was not required. Patients with a history of other malignancies within 5 years, except for basal or squamous cell carcinoma of the skin, were excluded.

### CTC analysis

**Fig. 1:**
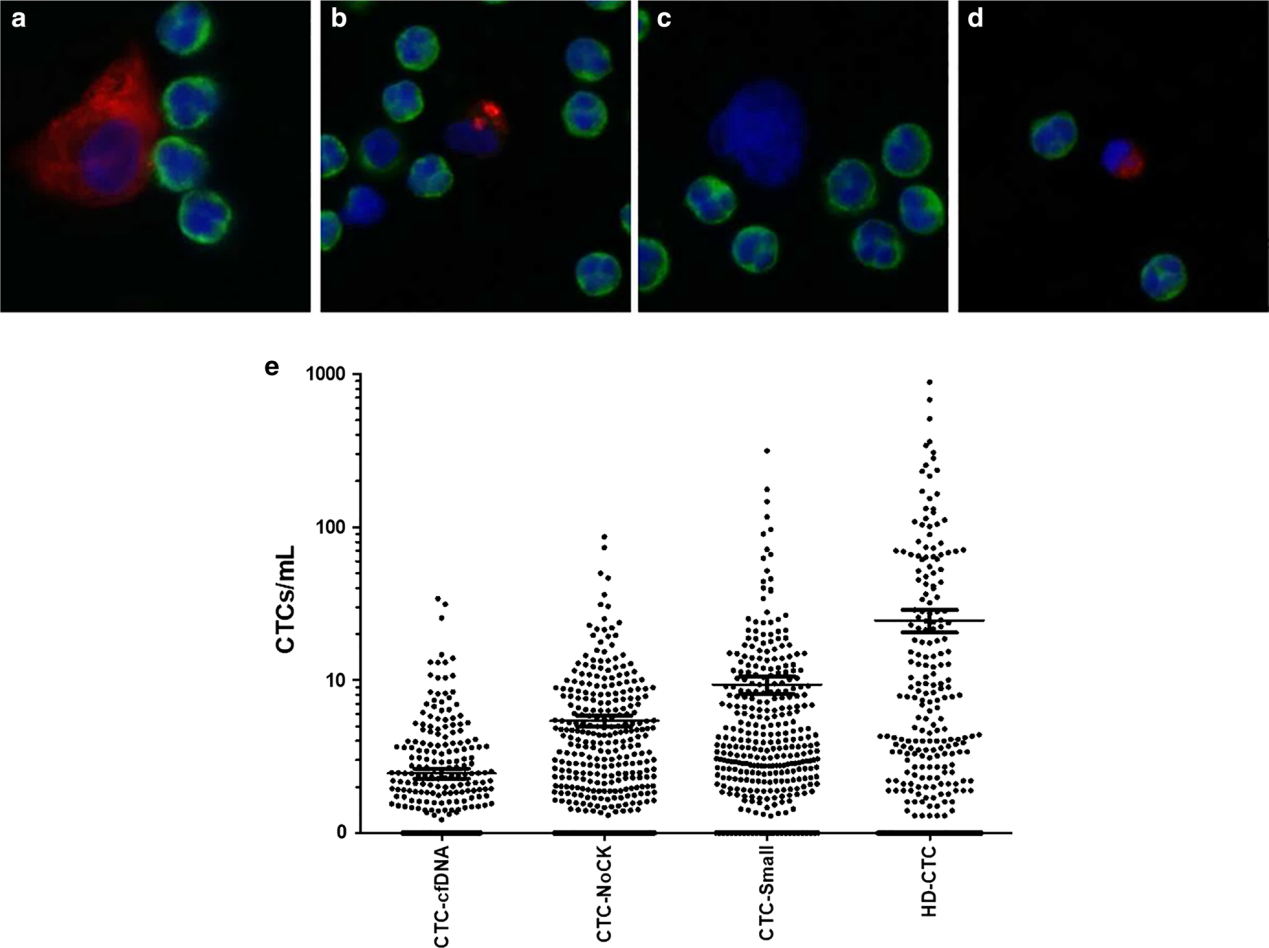
HD-CTC and candidate CTC cell data for stage IV NSCLC. **a** Representative image of HD-CTC. HD-CTCs are cytokeratin positive (red), CD45 negative (green), contains a DAPI nucleus (blue) and is morphologically distinct from surrounding white blood cells. **b**–**d** Representative images of types of suspected candidate CTCs found in a single NSCLC patient. **b** Nucleus too small and cytoplasm insufficiently circumferential; cell appears to be in late apoptosis and defined as CTC-cfDNA producing. **c** Suspected CTC that is negative for cytokeratin and CD45, but has a nucleus that is morphologically similar to CTCs, defined as CTC-NoCytokeratin. **d** Nucleus is small (same size as surrounding WBC nuclei) and cytokeratin present, defined as CTC-Small. **e** Distribution of CTCs and candidate cells in NSCLC patients

7.5 mL of blood was collected in a preservative tube (Cell-Free DNA BCT, Streck; Omaha, NE) prior to administration of chemotherapy. CTCs were monitored as previously discussed [22–27]. The sensitivity and accuracy of this assay was described previously [23, 27, 28]. Performance of the assay in healthy donors was also previously established. Each test for the detection of candidate cells consisted of analysis of an average of 4.2 million nucleated cells. In brief, following red blood cell lysis nucleated cells were attached as a monolayer to custom-made glass slides. For CTC identification, slides were incubated with antibodies against cytokeratin (CK; 1:100; Sigma, St. Louis MO) followed by an Alexa Fluor 555 secondary (Invitrogen, Carlsbad CA) and Alexa Fluor 647 conjugated anti-CD45 (1:125; AbD Serotec, Raleigh NC). Nuclei were counterstained with DAPI. Images of CTC candidates were presented to a hematopathologist-trained technical analyst for analysis and interpretation. Cells detected by the HD-SCA workflow were classified as HD-CTCs using the following criteria: CK^+^CD45^-^, intact DAPI nucleus without identifiable apoptotic changes or a disrupted appearance, and morphologically distinct from surrounding white blood cells (WBC; Fig. 1a) [22, 24–26]. WBC counts of whole blood were determined automatically (Medonic M-series Hematology Analyzer, Clinical Diagnostic Solutions Inc., Fort Lauderdale, FL) and the number of leukocytes detected by the assay per slide was used to calculate the actual amount of blood analyzed per test. Thus, fractional values of HD-CTCs/mL are possible.

Previous experimentation was conducted to ensure that CTC enumeration per slide analyzed was reliable and reproducible given the blood volume utilized. Depending on the WBC count of the blood sample, anywhere between 0.2–1.0 mL/slide is present. In an analysis of 130 blood samples from an independent NSCLC cohort, 4 slides per sample (approximately 10 million cells/sample) were analyzed by the HD-SCA workflow (Additional file 1: Figure S1 and Additional file 2: Table S1; Additional file 3: Table S2). Statistical analysis of 4 slides compared to any combination of 2 slides was not significantly different. Additionally, there was a significant correlation (Spearman r) between the 4 slide analysis and any combination of 2 slide analysis (Spearman r > 0.9300; P-value < 0.0001). This indicates that doubling the volume of blood analyzed, does not significantly affect the CTC enumeration per milliliter of blood.

The CTC population was morphologically heterogeneous within and across patients, varying in size, shape, and CK intensity. Representative cells that were tracked but not classified as HD-CTCs due to a lack of essential features based on either immunophenotype or morphology are also shown in Fig. 1. Cells that are CD45^−^CK^+^ and show cytoplasmic and/or nuclear apoptosis were defined as “CTC-cfDNA producing” (Fig. 1b). Cells that are CD45^−^ and express little to no CK, but otherwise meet the morphological criteria for CTCs are “CTC-NoCytokeratin” (Fig. 1c). Lastly, a subset of CD45^−^CK^+^ whose nuclear size was small (similar to the surrounding WBCs) are termed “CTC-Small” (Fig. 1e). It is important to note that these additional subtypes of cells may contribute to tumor dissemination.

### Statistical analysis

Statistical analyses were performed using RStudio (RStudio Team, 2015. RStudio: Integrated Development for R. RStudio, Inc., Boston, MA). Groups were compared using the Kruskal–Wallis Test, Welch’s t-test, or Wilcoxon signed-rank test, as appropriate. Correlation was analyses using the Pearson’s product-moment correlation test or Chi Square test. Kaplan–Meier analyses and the log-rank test were used for survival analyses. Overall survival (OS) time was calculated from the baseline blood draw date taken before initiation of a new line of therapy to the date of death. Progression free survival (PFS) time was calculated from the time of blood draw to the date of documented progression. Patients who were alive and progression free at last follow-up were censored. A p-value of ≤ 0.05 was considered significant.

## Results

### Patient demographics

A total of 81 patients who entered the study at the time of diagnosis (n = 41) or start of a next line of therapy (n = 40) with samples drawn at initiation and attempted redraws at 3 weeks, 3 months, and every 3 months thereafter until progression are reported here. Only 25 of these patients completed the study with at least one follow-up sample taken within 3 months of start of therapy and are subsequently used to understand the cellular dynamics of CTCs in this context of disease with a total of 117 samples. Patient demographics are described in Table 1.

**Table 1:** Patient demographics of stage IV NSCLC cohort

	NSCLC	Kinetics
	Cohort (n = 81)	Cohort (n = 25)
Age (years), median (range)	61 (33–90)	64 (39–78)
WBC count (e^6^), median (range)	6.8 (0.8–29.9)	8.1 (3.5–19.9)
Sex, n (%)		
Female	37 (45.7)	11 (44.0)
Male	32 (39.5)	13 (52.0)
N/A	12 (14.8)	1 (4.0)
Histologll, n (%)		
ACA	66 (81.5)	20 (80.0)
Sec	8 (9.9)	4 (16.0)
LCC	1 (1.2)	–
NOS	3 (3.7)	1 (4.0)
N/A	3 (3.7)	–
Location of primary mass, n (%)		
RUL	26 (32.1)	7 (28.0)
LUL	22 (27.2)	8 (30.0)
RML	3 (3.7)	–
RLL	13 (16.0)	3 (12.0)
LLL	12 (14.8)	2 (8.0)
R Hilum	1 (1.2)	1 (4.0)
None	2 (2.5)	1 (4.0)
N/A	2 (2.5)	3 (12.0)
Stage, n (%)		
IVA	18 (22.2)	6 (24.0)
IVB	40 (49.4)	18 (72.0)
Recurrent metastatic		1 (4.0)
IVA	5 (6.2)	–
IVB	13 (16.0)	–
N/A	5 (6.2)	–
Therpay, n (%)		
Entering 1st line	75 (92.6)	24 (96.0)
Crizotinib	1 (1.2)	1 (4.0)
Erlotinib	9 (11.1)	2 (8.0)
Platinum2	35 (43.2)	14 (56.0)
Platinum2 + erlotinib	1 (1.2)	–
Platinum3	28 (34.6)	7 (28.0)
Pemetrexed	1 (1.2)	–
Entering 2nd line	48 (59.3)	12 (48.0)
Crizotinib	2 (2.5)	1 (4.0)
Docetaxel	1 (1.2)	–
Erlotinib	37 (45.7)	8 (32.0)
Navelbine	1 (1.2)	–
Platinum2	3 (3.7)	2 (8.0)
Platinum3	3 (3.7)	1 (4.0)
Taxotere	1 (1.2)	–
Entering 3rd line	21 (25.9)	5 (20.0)
Crizotinib	1 (1.2)	–
Docetaxel	1 (1.2)	–
Erlotinib	10 (12.3)	4 (16.0)
Gemcitabine	1 (1.2)	–
Navelbine	1 (1.2)	–
Platinum2	5 (6.2)	1 (4.0)
Platinum3	1 (1.2)	–
Pemetrexed	1 (1.2)	–
Entering 4th line	18 (22.2)	2 (8.0)
Crizotinib	1 (1.2)	–
Erlotinib	4 (4.9)	1 (4.0)
Navelbine	10 (12.3)	1 (4.0)
Platinum2	2 (2.5)	–
Other	1 (1.2)	–
Entering 5th line	5 (6.2)	–
Navelbine	4 (4.9)	–
Platinum2	1 (1.2)	–
Entering 6th line	–	–
Erlotinib	2 (2.5)	–
Entering 7th line	–	–
Navelbine	1 (1.2)	–
Entering 8th line	–	–
Etoposide	1 (1.2)	–
Site of secondary metastatic lesions, n (%)		
Adrenal gland	8 (9.9)	4 (16.0)
Bone	32 (39.5)	10 (40.0)
Brain	21 (25.9)	6 (24.0)
Liver	14 (17.3)	3 (12.0)
Pleura	29 (35.8)	6 (24.0)
Lung	28 (34.6)	8 (32.0)
Kidney	1 (1.2)	–
Pericardium	3 (3.7)	–
Muscle	1 (1.2)	1 (4.0)
Skin	2 (2.5)	1 (4.0)
Axillary LN	3 (3.7)	–
Spleen	1 (1.2)	–
Number of distant metastatic lesions, n (%)		
0	23 (28.4)	8 (32.0)
1	37 (45.7)	11 (44.0)
2	19 (23.5)	3 (12.0)
3	20 (2.5)	3 (12.0)
Mutation, n(%)		
ALK		
Positive	2 (2.5)	1 (4.0)
Negative	3 (3.7)	2 (8.0)
KRAS		
WT	12 (14.8)	3 (12.0)
Mutant	6 (7.4)	4 (16.0)
EGFR		
WT	10 (12.3)	–
Mutant	7 (8.6)	–

ACA: adenocarcinoma; SCC: squamous cell carcinoma; LCC: Large-cell carcinoma; NOS: Not otherwise specified; RUL: right upper lobe; LUL: left upper lobe; RML: right middle lobe; RLL: right lower lobe; LLL: left lower lobe; LN: lymph node; N/A: data not available

### CTC prevalence

HD-CTCs demonstrated heterogeneous morphological features as previously described for both NSCLC and other carcinomas [22, 24–26, 29–31], with cell shapes and sizes varying within and across patients. Multiple peripheral blood samples were collected at various timepoints per patient. A total of 362 samples across a total of 139 lines of therapy from 81 patients were analyzed using the HD-SCA workflow. Sample positivity was defined as > 0 CTC/mL [22]. Overall 209 (57.74%) separate samples were determined to be HD-CTC positive.

At baseline, 51 (62.96%) patients were positive for HD-CTCs. HD-CTC levels in NSCLC patients at baseline ranged from 0 to 509.20, with a median of 2.20 (mean 26.21 ± 15.64) HD-CTCs/mL (Fig. 1e). There was no significant correlation between the baseline CTC count and patient clinicopathological characteristics. The HD-CTC count did not discriminate between primary tumor histology, which is consistent with other advanced NSCLC studies [24, 32–35]. There was no correlation between stage including the presence or absence of metastatic (M1) disease and HD-CTC/mL. There was a significant difference in the change of CTCs/mL from baseline draw to the end of study when stratifying patients by M status (p-value = 0.0230, Wilcoxon rank sum test), indicating that CTC levels in M1b patients are less dynamic over time. This suggests that the observable difference in temporal-linked cellular enumeration may be more related to patient disease characteristics than simply baseline enumeration of HD-CTCs.

Next we explored the predictive capacity of HD-CTCs of as a biomarker for differential treatment by examining 28 patients who received bevacizumab-based triplet regiments. For 5 of the bevacizumab treated patients, we did not have follow-up blood samples. The remaining 23 patients receiving bevacizumab had an increase in HD-CTCs at the time of the second blood draw (p-value = 0.0029). At the first follow-up blood draw post initiation of treatment, bevacizumab treated patients (33.3%) had a median of 11.70 (range 0–885.10, mean 77.69 ± 83.52) HD-CTCs/mL, while patients receiving an alternative therapy had a median of 0.70 (range 0–677.60, mean 29.17 ± 32.35) HD-CTCs/mL. Over the course of the study, there was a significant decrease in HD-CTCs/mL from baseline to the last blood draw in patients receiving bevacizumab treatment (Wilcoxon test, p-value = 0.0196). This indicates that HD-CTCs are highly dynamic during the course of treatment, and that the time frame analyzed is critical in understanding response to therapy and disease progression.

The HD-SCA workflow identifies rare cells not typically found in healthy individuals [23] based on cellular size or biomarker (CK and CD45) expression. The distribution of other defined cellular events that do not meet the inclusion criteria for HD-CTC is depicted in Fig. 1e, with descriptive statistics provided in Table 2. Rare cells of any type were detected in 97% of the samples. The other candidate cell types all presented at significantly variable levels (p-values < 0.002). Total HD-CTC count was significantly higher than that of CTC-Small (p-value = 0.0006) and CTC-cfDNA producing cells (p-value < 1.2710e^−9^). There was not a statistically significant difference between HD-CTC and CTC-NoCytokeratin counts. There was a positive association between overall HD-CTC count and enumeration of CTC-Small (p-value = 0.0010, Person’s correlation = 0.1718), CTC-NoCytokeratin (p-value = 0.0016, Person’s correlation = 0.1649), and CTC-cfDNA producing (p-value = 2.41e^−5^, Person’s correlation = 0.2200) cells. There was also a positive correlation between the quantification of CTC-NoCytokeratin cells and CTC-Small cells (p-value = 4.15e^−6^, Person’s correlation = 0.2393) and CTC-cfDNA producing cells (p-value = 6.53e^−8^, Person’s correlation = 0.2793). These additional cellular events were monitored as CTC candidate cells in an attempt to understand their pathogenic contributions. One advantage of a non-biased workflow, in which no cell is left behind, is the ability to observe all rare cells in the liquid biopsy, allowing us to study the role of each in the context of NSCLC. These non-traditional CTCs would be missed using an approach that requires enrichment.

**Table 2:** Descriptive statistics for the distribution of CTCs and candidate cells in stage IV NSCLC patients

	CTC-cfDNA	CTC-NoCK	CTC-Small	HD-CTC
Minimum	0.000	0.000	0.000	0.000
25% percentile	0.000	0.417	0.917	0.000
Median	0.000	1.832	2.423	1.000
75% percentile	1.480	5.324	7.428	8.625
Maximum	33.14	85.53	314.4	885.1
Mean	1.454	4.423	8.353	23.61
Std. deviation	3.437	8.320	23.75	79.04
Std. error	0.1827	0.4422	1.262	4.154
Lower 95% Cl	1.095	3.554	5.871	15.44
Upper 95% Cl	1.813	5.293	10.83	31.78

### Survival analysis

**Fig. 2:**
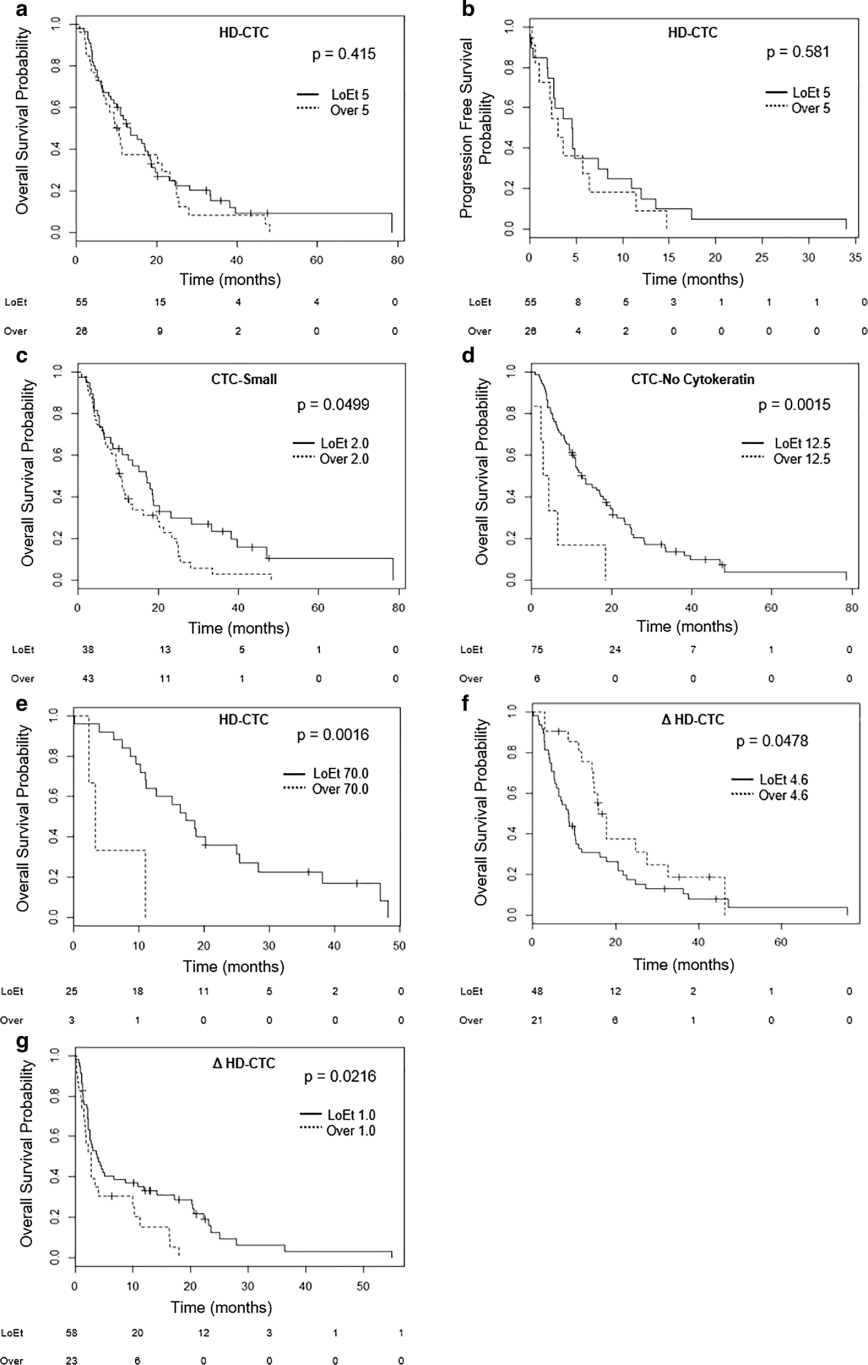
Kaplan-Meier survival analysis of stage IV NSCLC cohort. **a** Baseline HD-CTC enumeration did not significantly affect OS. **b** Baseline HD-CTC enumeration did not significantly affect PFS. **c** Patients with > 2.0 CTC-Small cells/mL at baseline have a significantly shorter OS. **d** Patients with > 12.5 CTC-NoCytokeratin cells/mL at baseline have a significantly shorter OS. **e** Patients receiving bevacizumab with > 70.0 HD-CTCs/mL at baseline have a significantly shorter OS. **f** Patients with > + 4.6 Δ HD-CTCs/mL from the first to second blood draw have a significantly shorter OS. **g** Patients with > + 1.0 Δ HD-CTCs/mL from the first to last blood draw have a significantly shorter OS. LoET: lesser or equal to. The number of at risk individuals per group is shows below each graph

The change in HD-CTC count between samples was strongly associated with survival, while the absolute numbers of HD-CTCs or other cell categories were only weakly associated with OS. The median OS for patients was 11.07 (95% CI 5.16–20.27) with a range of 0.03–78.56 months (mean 15.68 ± 3.08), while the median PFS was 0 (95% CI 0–2.56) with range 0–34 months (mean 2.34 ± 1.15). From Kaplan–Meier analysis for this data set the 1-, 2-, and 5-year OS for stage IV NSCLC patients was determined to be 49%, 25%, and 3%, respectively, consistent with average [36]. Figure 2 shows the Kaplan–Meier curves for OS and PFS in the 81 patients according to their CTC count. Interestingly the OS for 38 (47%) patients with ≤ 2.0 CTC-Small cells/mL was 17.07 (95% CI 6.18–31.42) months versus 10.22 (95% CI 5.04–19.17) months for patients with > 2.0 CTC-Small cells/mL (n = 43, 53%) at baseline blood draw (p-value = 0.0499; Fig. 2c). When looking at the CTC-NoCytokeratin cells, the 75 (93%) patients with ≤ 12.5 cells/mL had a median OS of 11.76 (95% CI 6.37–22.26) months compared to 3.58 (95% CI 2.47–5.92) months for the 6 (7%) patients with > 12.5 CTC-NoCytokeratin cells/mL (p-value = 0.0015; Fig. 2d). CTC-cfDNA producing cell number was not a significant predictor of OS or PFS. Additionally, baseline HD-CTC count was not predictive of OS or PFS (Fig. 2a, b).

Further analysis was used to evaluate the association of CTCs and combinational first line therapy with bevacizumab. Baseline HD-CTC count was a predictor of survival for patients on bevacizumab treatment. Looking at the 28 (35%) patients receiving bevacizumab, the 3 (11%) patients with > 70.0 HD-CTC/mL at baseline had a significantly shorter OS (p-value = 0.0016; Fig. 2e) with a median of 3.35 (95% CI 2.84–7.15, range 2.33–10.94) and a mean of 5.54 (± 11.68) months. Patients with ≤ 70.0 HD-CTCs/mL at baseline with bevacizumab treatment had an OS median of 17.18 (95% CI 10.19–25.43, range 0.03–48.23) and mean of 19.94 (± 5.59) months. The 6-month survival probability of patients that received bevacizumab with a baseline HD-CTC count ≤ 70.0 cells/mL was 92%, while the 1- and 2-year survival probabilities were 64% and 36%, respectively. Those patients receiving bevacizumab with > 70.0 HD-CTCs/mL at baseline had a 6-month survival probability of 33% and all succumbed to disease within 1 year. Interestingly this HD-CTC threshold was not significant in the whole cohort, but specific to bevacizumab treated patients, indicative of potential prognostic utility of baseline enumeration as a companion diagnostic for differential treatments.

The change in HD-CTC counts (HT-CTC Kinetics) between samples during the course of therapy was determined to be a strong predictor of OS. The 48 (70%) patients with ≤+4.6 ΔHD-CTCs/mL at the first follow-up blood draw had a median OS of 8.64 (95% CI 4.36–18.45, range 0.16–75.80) months which was significantly different than the 15.77 (95% CI 11.89–24.77, range 2.89–46.29) months for the 21 (30%) patients with > + 4.6 ΔHD-CTCs/mL (p-value = 0.0478; Fig. 2f). For the 58 (72%) patients with > + 1.0 ΔHD-CTCs/mL from baseline to the time of last blood draw, the median OS was 2.76 (95% CI 1.30–8.15, range 0.16–17.97) months and was significantly shorter than the 3.71 (95% CI 1.62–13.85, range 0.03–54.87) months for the 23 [28] patients with ≤ + 1.0 ΔHD-CTCs/mL (p-value = 0.0216; Fig. 2g). Changes in HD-CTC enumeration from repeat samples was not predictive of PFS at any threshold for all draw numbers (data not shown). Together this indicates that the CTC kinetics between baseline and follow-up samples, rather than a single time point enumeration, is more clinically applicable as a prognostic tool for survival.

### Exploring CTC Kinetics in a subset of NSCLC patients

#### Patient demographics

The HD-CTC Kinetics were examined in 25 patients with stage IV NSCLC that had a baseline sample collected at the start of either the 1st, 2nd, or 3rd line of therapy with at least one follow-up sample taken within 3 months. Patient demographics are presented in Table 1. The demographics of the kinetic subset were similar to that of the whole cohort.

#### CTC prevalence and dynamics within 3 months of initiation of chemotherapy

**Fig. 3:**
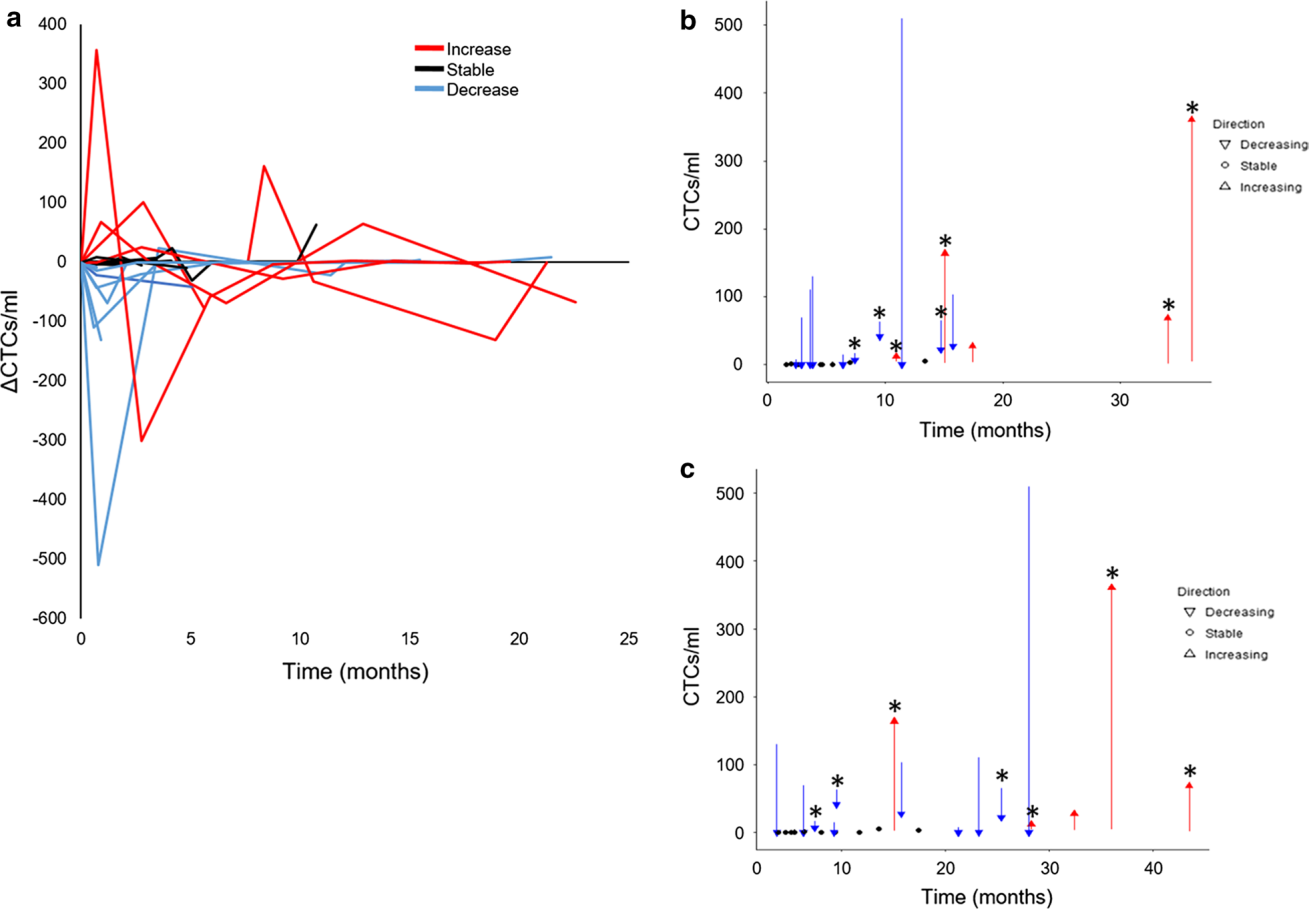
HD-CTC enumeration data for the Kinetics cohort (n = 25). **a** Change in HD-CTC counts in NSCLC patient samples after entering 1st line therapy. **b**, **c** HD-CTC levels before and after entering 1st line therapy where arrows show the direction and magnitude of change and circles represent patients with stable HD-CTC levels. Patients that received bevacizumab treatment are shown with an asterisk. Graphical representation of PFS (**b**) and OS (**c**) are provided. Patients with (red) increasing levels, (black) stable levels, and (blue) decreasing levels of HD-CTCs/ml

HD-CTCs were detected in 21 of 25 cases (84%) at initiation of therapy with a median of 3.70 (range 0–509.20, mean 40.79 ± 42.95) HD-CTCs/mL. Eight (32%) patients had > 10 and 3 (12%) patients had > 100 HD-CTCs/mL before the start of therapy. Additional blood samples were collected approximately 3 weeks and 3 months post start of a new line of therapy. Twelve (48%) patients had both subsequent blood draws, while 13 (52%) patients had only 1 sequential blood sample taken. HD-CTC Kinetics in patients undergoing chemotherapy were shown to follow 3 distinct patterns as determined by the first 3 months: an increase in HD-CTCs numbers, unchanged HD-CTCs numbers (stable), and a decline in HD-CTCs numbers with therapy (Fig. 3a). HD-CTC stability was defined as less than a 5.0 HD-CTCs/mL change from baseline. Five (20%) patients had an increasing HD-CTC count (median 3.7, range 1.7–4.6, mean 3.26 ± 1.10 HD-CTCs/mL); 10 (40%) patients had a decrease (median 63.9, range 3–509.2, mean 98.78 ± 93.37 HD-CTCs/mL); and 10 (40%) patients had HD-CTC numbers that remained unchanged (median 0.9, range 0–4.7, mean 1.56 ± 1.08). All patients showing an increase or decrease in HD-CTCs were CTC positive at baseline, while only 6 (24%) patients with stable HD-CTC levels were CTC positive at baseline.

To further assess the relationship between HD-CTC Kinetics and prognosis, the direction and magnitude of the change in HD-CTC enumeration from baseline in the first 3 months on new therapy were plotted against the time of survival (Fig. 3b, c). Patients that experienced a rise in HD-CTC number had a median increase of 67.4 (range 8.1–356.0, mean 123.74 ± 125.20) HD-CTCs/mL. Patients with stable disease presented with a median change of 0 (range − 4.7 to 2.3, mean 0.54 ± 1.35) HD-CTCs/mL. In the subgroup of patients that had a reduction in HD-CTC number during the first 3 months, the median decrease was 55.95 (range 7.8–509.2, mean 99.09 ± 93.18) HD-CTCs/mL. Patients that received bevacizumab treatment (n = 7, 28%) did not have an observable pattern of HD-CTC kinetics.

**Fig. 4:**
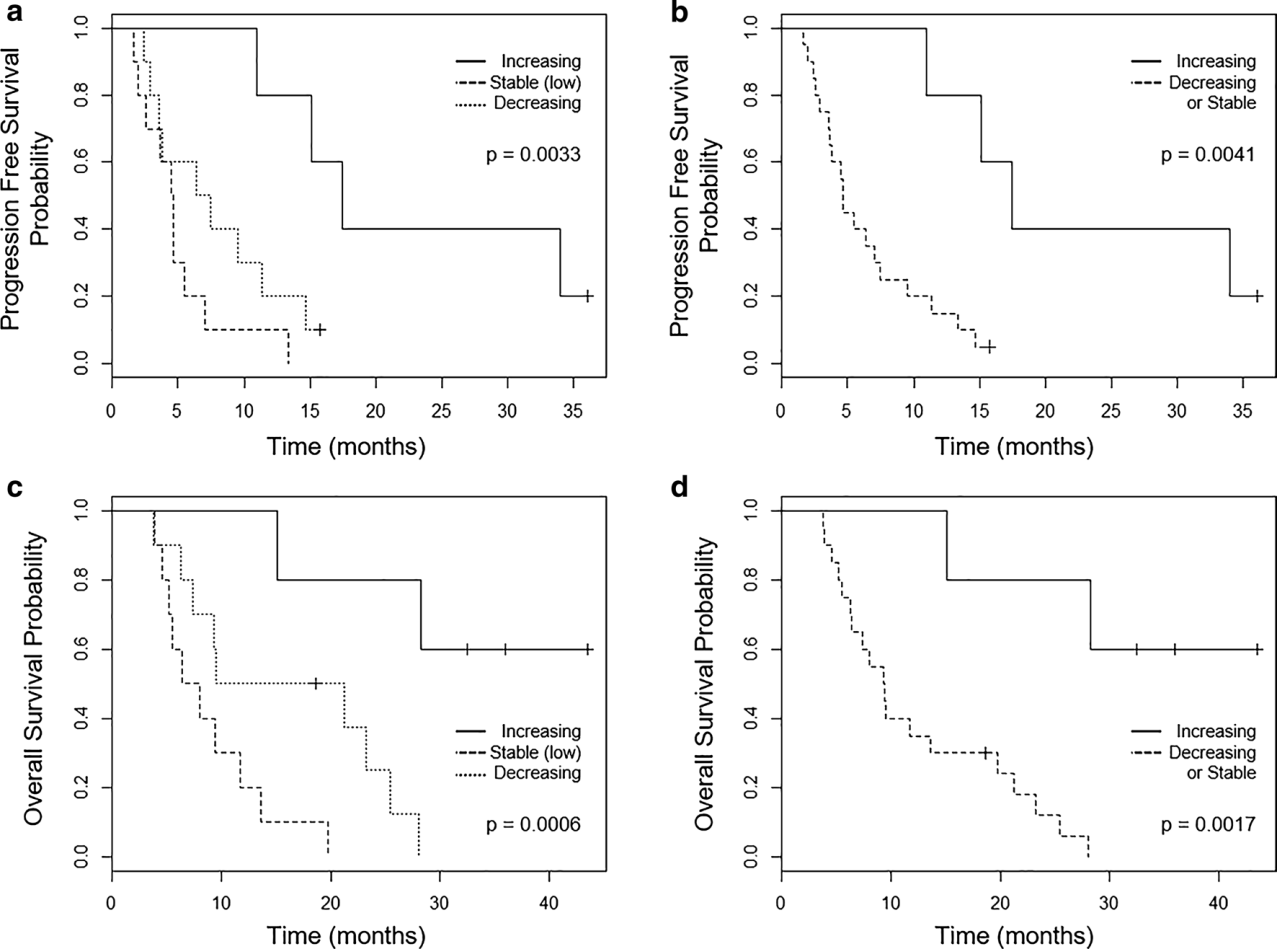
Kaplan-Meier analysis of OS (**a**, **b**) and PFS (**c**, **d**) for stage IV NSCLC kinetic cohort. Patients are stratified according to HD-CTC dynamics, into three groups (**a** and **c**): increase, stable, or decrease, or into two groups (**b** and **d**) where stable and decreasing patients are combined. Significant benefit in terms of increased OS and PFS for patients displaying an increasing number of HD-CTCs (P-value < 0.05)

The kinetics cohort had an overall median PFS of 6.41 (95% CI 3.61–13.37, range 1.61–36.04, mean 9.63 ± 3.73) months. There was a significant difference in PFS between the distinct dynamic HD-CTC profiles (Fig. 4a, b). Patients with an increasing HD-CTC profile had a median PFS of 17.41 (95% CI 15.08–34.00, range 10.94–36.04, mean 22.70 ± 14.29) months. The stable profile had a median PFS of 4.57 (95% CI 2.83–5.27, range 1.61–13.37, mean 4.94 ± 2.43) months, while the decreasing profile had a median PFS of 6.92 (95% CI 3.64–10.93, range 2.37–15.77, mean 7.79 ± 3.51) months. Patients with an increasing HD-CTC profile has a significantly longer PFS compared to stable and decreasing HD-CTC profiles (p-value = 0.0040 and 0.0080, respectively). There was not a significant difference in PFS between stable and decreasing HD-CTC profiles. The 1-year progression free survival probability for patients with increasing, decreasing, or stable HD-CTC profiles was 80%, 20%, and 10% respectively. PFS was shown to be significantly associated with CTC dynamics at the initiation of new therapy.

Baseline HD-CTC number did not have a significant association with survival in this small cohort, a finding similar to the full study subset. For all patients, the median OS was 11.76 (95% CI 6.41–23.26, range 3.81–43.50, mean 15.66 ± 4.63) months. By subcategorizing patients into one of the distinct dynamic CTC profiles observed within the first 3 months of new therapy, there was an observable significant difference in OS (Fig. 4c, d). Patients with increasing HD-CTC counts exhibited a significantly longer OS compared to both stable and decreasing groups (p-value = 0.00003 and 0.0089, respectively). Patients with increasing HD-CTC count had a median OS of 32.46 (95% CI 28.29–36.04, range 15.11–43.50, mean 31.08 ± 13.07) months. Patients with stable and decreasing HD-CTC count had a median OS of 7.23 (95% CI 5.24–11.18, range 3.91–19.75, mean 8.59 ± 3.19) and 12.65 (95% CI 7.89–22.76, range 3.81–28.06, mean 15.12 ± 6.34) months respectively. There was a significant difference between OS for patients with stable and decreasing HD-CTC profiles (p-value = 0.0397). The overall 1-year survival probability for patients was 100%, 50%, and 20%, presenting with an increase, decreasing, or stable in HD-CTC number, respectively. The 2-year overall survival rate for patients with an increase in HD-CTC count was 80%, while those with a decrease in HD-CTC number was 25%. All patients with stable HD-CTC count succumbed to disease within 2 years. Therefore patients with an increasing CTC counts during the first 3 months post initiation of treatment have a better PFS and OS, indicating that the cellular dynamics are critical in prognostic evaluations.

## Discussion

Detection and characterization of CTCs from the liquid biopsy is a powerful diagnostic and prognostic tool for cancer patients, providing insight into the potential of therapy failure. The HD-SCA technology is a validated rare cell detection workflow that has joined forces with other technologies to build the Blood Profiling Atlas Commons (Blood PAC) by standardizing methods for analysis of circulating tumor cell morphology, genomics and proteomics, with simultaneous cell-free genomics. The goal of BloodPAC is to provide the scientific knowledge so physicians and patients are more informed about their disease and can make better-informed decisions. Recently Epic Sciences, using a very similar methodology as presented here, released the only predictive and prognostic test for the detection of CTCs positive for nuclear-localized androgen receptor splice variant 7 (AR-V7) in metastatic castration-resistant prostate cancer (mCRPC). The Epic AR-V7 assay shows that detection of even one CTC with nuclear AR-V7 positivity translates to resistance to enzalutamide and abiraterone and sensitivity to the taxanes in mCRPC [37–39]. This supports our data due to the utilization of analogous technology, justifying further study into the clinical utility of the HD-SCA workflow for the identification of a biomarker in NSCLC patients. The other FDA approved CTC detection technology (CellSearch^®^) involves enrichment for EpCAM, with subsequent identification of CTCs using CK expression. In a direct comparison to CellSearch^®^, the HD-SCA workflow detects significantly more CTCs in a higher proportion of patients with higher sensitivity; while healthy donor samples present with ≤ 1 CTC/mL [23]. Various enrichment and identification methods lead to a variety of test results and different sensitivities and/or specificities. Only through use of a non-EpCAM based approach, such as the HD-SCA workflow, can all potential CTCs be monitored, including EpCAM-low or negative cells.

The term ‘liquid biopsy’ refers to analyses typically performed on peripheral blood collected from a patient, but each technological platform produces different results due to inherent design components optimizing evaluation of different analytes. At baseline 63% of our patients had HD-CTCs, while overall 58% of the total samples collected during this longitudinal study were positive. Of the studies that have evaluated advanced NSCLC, only a few have reported a similar baseline detection rate [24, 32]. Over the past years exciting technologies have been developed based on various physical and biological properties. Using CellSearch^®^, Punnoose et al. detected CTCs in 78% of samples [8], while other studies using that platform have shown much lower detection rates of 23% [6], 36.4% [15], 41.9% [19], 32% [20], and 30.9% [21]. Higher detection rates have been reported for NSCLC using a magnetic sifter (100%) [40], vortex chip (100%) [41], DFF (100%) [42], MCA (77%) [20], Cyttel (87%) [35], CanPatrol CTC enrichment (79%) [43], and multi-immunofluorescence staining (100%) [44].

Multiple studies have indicated that baseline CTC counts are prognostic in NSCLC [7, 15, 16, 19, 24, 34, 35, 45], but the prognostic significance of CTC dynamics over the course of treatment is still unclear and may provide more utility in clinical management of metastatic lung cancer. Patient management would be revolutionized if through monitoring the dynamics of CTCs, patients could be classified into prognostic groups with specific treatment plans to alter the course of disease, i.e. predictive classifications. Krebs et al. [7] has reported a threshold of CTC levels that was prognostic, and for patients positive at baseline an increasing CTC level was associated with poor prognosis and a lack of response. Punnoose et al. [8] also showed that an increase in CTC counts after treatment was associated with a poor response, but that absolute CTC levels were not prognostic. Both of these studies utilized the CellSearch^®^ platform for CTC detection and enumeration. Zhang et al. [35] analyzed 46 advanced NSCLC patients using the Cyttel method, showing CTC dynamics were not correlated to RECIST evaluation, but patients with an increasing CTC profile had either SD or PR. More recently He et al. [46] analyzed 66 stage IV NSCLC patients using flow cytometry, in which patients with effective treatment (60.6%) had a decrease in CTC count from baseline. Significant differences between these studies that may provide insight into the discordance of results include (i) different CTC detection methods, (ii) patient population disease stage, (iii) patient inclusion criteria, (iv) follow up period, and (v) definition of CTC increase/decrease.

A strong prognostic correlation of HD-CTC dynamics was identified in which patients displaying an increase in HD-CTCs after entering a new line of chemotherapy, typically within three months, do significantly better in terms of PFS and OS than patients who don’t exhibit that change. It should be noted that this analysis was not pre-specified and will require validation in an independent cohort. Although the association between an increase in HD-CTCs during therapy and prolonged PFS and OS may be counterintuitive, when considering the setting of metastatic disease as opposed to limited disease, it is unsurprising. The prognostic value of CTC enumeration alone may be largely lost in patients where metastatic disease has already been established. We further suggest that the improved survival outcomes seen in patients with increasing HD-CTC levels may be the result of chemotherapy effectively acting in the tumor tissue, causing it to shed cells into the circulation. This is corroborated by the fact that all patients with increasing HD-CTC levels shortly after initiation of treatment show clinical response or stable disease according to the assessment of PFS. This demonstrates that CTC kinetics over time are more relevant than enumeration at a single timepoint, implying that CTC prognostic ability is dependent on the specific context of use.

To better understand the prognostic significance of CTCs for differential treatments, we looked at those patients specifically being treated with bevacizumab. The monoclonal antibody against VEGF, bevacizumab, has been shown to improve OS in NSCLC when combined with chemotherapy [47]. It is well recognized that bevacizumab, through the inhibition of tumor angiogenesis, leads to intra-tumoral hypoxia triggering tumor progression, invasive cell behavior, and EMT [48]. Because the HD-SCA technology is independent of EpCAM expression it is uniquely positioned to be able to evaluate CTC fluctuations on bevacizumab therapy. In the small cohort of bevacizumab treated NSCLC patients, we showed that a baseline threshold of HD-CTC enumeration was prognostic of survival. Unfortunately the small sample size in the kinetics cohort limits our statistical analysis of cellular dynamics, warranting further study. Previous studies have shown a decrease in CTC count from baseline in breast cancer patients [49] and in colorectal cancer [50, 51] receiving bevacizumab. It is reasonable to think that different treatments affect the disease and therefore the CTCs in unique ways, suggesting that CTC prognostic ability may be therapeutic dependent. This would imply the need to develop a cellular signature specific to the treatment type. Further research will be vital in understanding the relationship between CTCs and clinical response to specific treatments.

In a previous publication utilizing the HD-SCA workflow, Nieva et al. [24] showed detection of HD-CTCs in 68% of samples from patients with NSCLC and this is consistent with our finding in this follow up study. Nieva et al. [24] averaged all CTC counts from multiple blood draws for each individual patient to show a survival difference with a threshold of 5 CTCs/mL. Conducting a similar analysis on the cohort presented here confirms that higher numbers of detected CTCs were associated with poor PFS (Additional file 4: Figure S2). Survival analysis for the study presented here was conducted in a more finely temporally nuanced method, utilizing each individual blood draw as well as the change in CTC dynamics, wherein each draw is dependent on the previous to show the effects of cellular kinetics. Additionally, here we emphasize the time of sample collection as key to understanding the cellular changes, in which data from the specific timepoint of 3 months post start of treatment was analyzed, as compared to our colleagues’ analysis which used all samples, include those collected at 180 + days post baseline draw.

The primary tumor, CTCs, and metastatic lesions show considerable heterogeneity in cellular morphology. Depending on the CTC criteria used for each detection platform, specific subpopulations of CTCs are identifiable. In a meta-analysis conducted by Wang et al. [52] different CTC subpopulations were determined to be of predictive clinical value. Using the HD-SCA workflow various candidate cells are monitored, but are not defined as classic CTCs. The key issue is whether these different subtypes of circulating cells reflect the primary tumor or if they are distinct. Muinelo-Romay et al. [19] enrolled 43 newly diagnosed NSCLC patients, and found a statistically positive correlation between intact CTCs and apoptotic CTC or CK fragments using CellSearch^®^. The data presented here also indicates a correlation between HD-CTCs and candidate CTC cells identified by HD-SCA, indicative of a common origin. Taken together this demonstrates that the various candidate cells detected by each platform are subgroups of CTCs which may have prognostic significance. This is reflected here by the observable shorter OS probability with higher baseline cell counts of CTC-Small and CTC-NoCytokeratin cells. Interestingly, this also suggests that morphologically distinct cells lacking an epithelial biomarker may be critical in predicting patient outcomes. This is supported by the work from Nel et al. [44] showing an association between mesenchymal CTCs and poor response, which is also confirmed by the Yin et al. [53] study in which EpCAM negative CTCs were associated with shorter survival. In optimizing CTC detection for clinical utility, it will be crucial to monitor all subpopulations of CTCs for identification of specific cells driving metastasis and chemotherapeutic resistance, while also obtaining a complete representation of the tumor heterogeneity.

Not all CTCs may be tumor initiating cells, but may indeed be a good biomarker for response. CTCs may be the result of either spontaneous release or as a direct result of medical procedures (biopsy/surgery). In specific cases a simple ‘CTC yes’ vs. ‘CTC no’ may be prognostics, especially when determining the best therapy. For some patients, ‘CTC yes’ may suggest a more aggressive treatment plan is needed. Other patients with ‘CTC no’ may do well on a less aggressive treatment plan, possibly sparing them side effects. Absolute CTC count beyond this scenario has not been proven clinically meaningful. The use of CTC kinetics may be clinically useful in monitoring response and survival of individual patients. Patient-specific CTC kinetic rates could be utilized as a companion tool to clinical imaging, allowing for better stratification of patients for specific therapeutics or early detection of treatment failure.

## Conclusions

The data presented here motivates the potential use of CTC kinetics for monitoring response to therapy and improving the ability to define prognosis. The scenarios that are most relevant would be (1) measurement of baseline CTC counts for patients entering bevacizumab treatment, (2) assessment of other candidate cell populations prior to treatment for prognostic insight, and (3) the use of CTC kinetics as a marker for progression to trigger imaging and/or additional molecular studies. This should motivate a larger, fully powered study with a larger cohort of stage IV NSCLC patients entering a new or first line of therapy and peripheral blood sample collections that are more frequent than the protocol used here. By understanding the dynamic processes of CTCs, we can monitor important functions or behaviors of the cellular network induced by changing conditions due to the initiation of treatment. The results presented open new avenues of investigation into the complex role of CTCs in cancer research and clinical management by suggesting that the level and dynamics of CTCs may indicate very different biological and pharmacological phenomena at different stages of disease and timepoints of treatment. This type of dynamic biomarker will become a key component in the evaluation of an end point in clinical trials to monitor efficacy of therapy by distinguishing responding from non-responding patients, facilitate the identification of new therapeutic targets, as well as expedite approval for novel anticancer therapies for NSCLC.

## Supplementary information

**Additional file 1: Figure S1.** HD-SCA analysis of 130 blood samples from an independent NSCLC cohort in which 4 slides/test were analyzed. A) HD-CTCs/mL for 2 or 4 slides, B) HD-CTC count per test of 2 or 4 slides, and C) correlation between 4 slides and any 2 slides. Left: all data; Right: values < 100. Analysis of 4 slides compared to any combination of 2 slides showed a lack of statistically significant difference (P-value > 0.05). Statistically significant correlation (Spearman r) between 4 slide analysis and any combination of 2 slide analysis (Spearman r > 0.9300; P-value < 0.0001) is shown.

**Additional file 2: Table S1.** CTC enumeration based on 4 slides compared to any combination of 2 slides using the HD-SCA workflow for 130 NSCLC liquid biopsy samples using the Wilcoxon matched-pairs signed rank test.

**Additional file 3: Table S2.** Correlation between CTC enumeration based on 4 slides versus 2 slides using the HD-SCA workflow for 130 NSCLC liquid biopsy samples (Spearman’s r correlation test).

**Additional file 4: Figure S2.** Kaplan–Meier survival analysis of PFS (A) and OS (B) of stage IV NSCLC cohort conducted similarly to Nieva et al, in which all CTC counts from multiple blood draws for each individual patient were averaged in an attempt to show a survival difference (24). This confirms the previously reported results that higher numbers of detected CTCs were associated with unfavorable prognosis in advanced NSCLC.

## Data Availability

The datasets used and/or analyzed during the current study are available from the corresponding author on reasonable request.

## Abbreviations

NSCLC: non-small cell lung cancer
CTC: circulating tumor cell
HD-SCA: high-definition single cell assay
CK: cytokeratin
WBC: white blood cells
PFS: progression-free
OS: overall survival
EpCAM: epithelial cell adhesion molecule
cfDNA: cell free DNA
AR-V7: androgen receptor splice variant 7
