# Supplementary figures and images for: Circulating tumor cells as a response monitor in stage IV non-small cell lung cancer
Source: J Transl Med. 2019 Aug 28;17:294. doi: 10.1186/s12967-019-2035-8 (PMC6714097; doi:10.1186/s12967-019-2035-8)

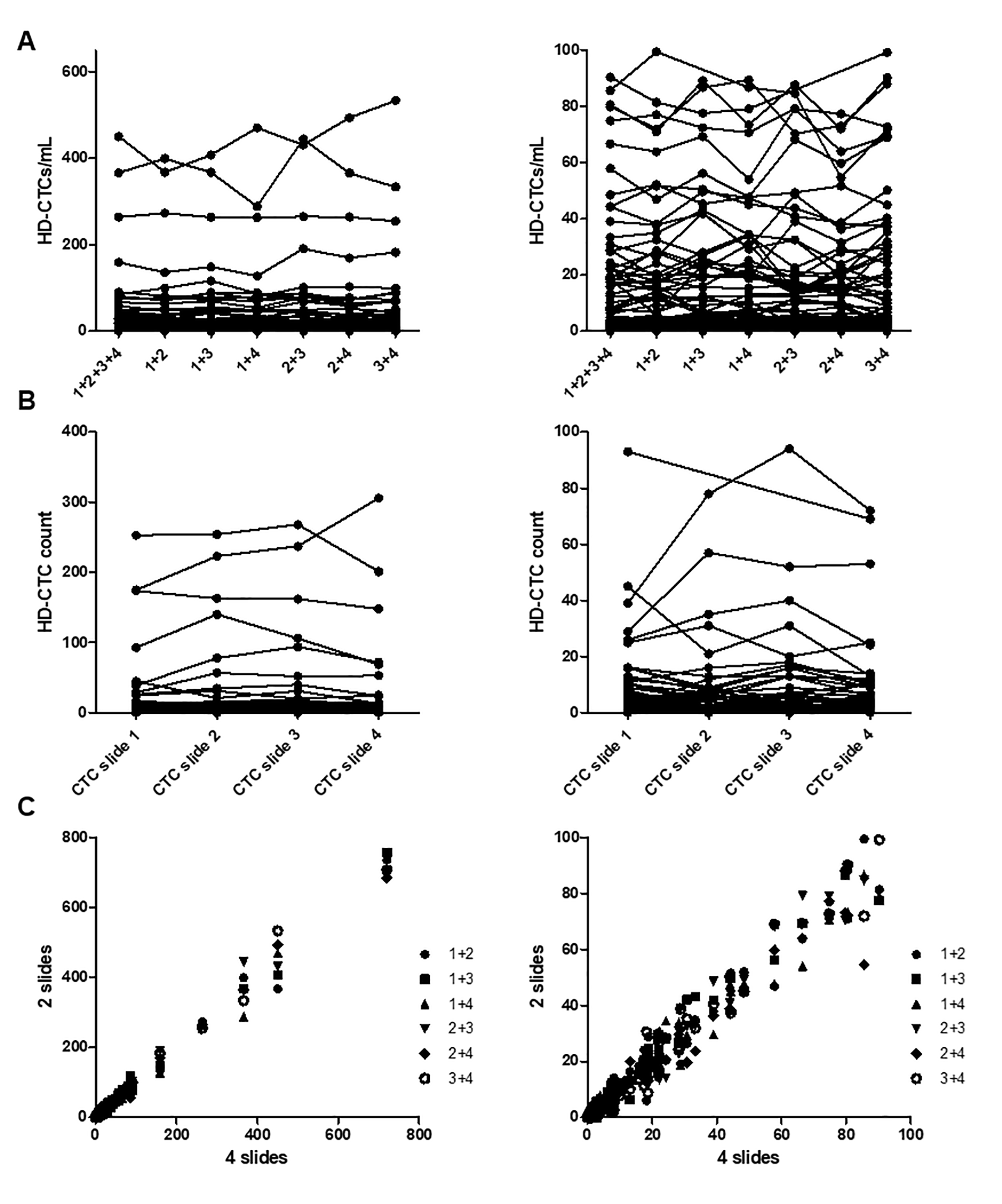

Supplement: Supplementary file 1 — Additional file 1: Figure S1. HD-SCA analysis of 130 blood samples from an independent NSCLC cohort in which 4 slides/test were analyzed. A) HD-CTCs/mL for 2 or 4 slides, B) HD-CTC count per test of 2 or 4 slides, and C) correlation between 4 slides and any 2 slides. Left: all data; Right: values < 100. Analysis of 4 slides compared to any combination of 2 slides showed a lack of statistically significant difference (P-value > 0.05). Statistically significant correlation (Spearman r) between 4 slide analysis and any combination of 2 slide analysis (Spearman r > 0.9300; P-value < 0.0001) is shown. [file 12967_2019_2035_MOESM1_ESM.tif]

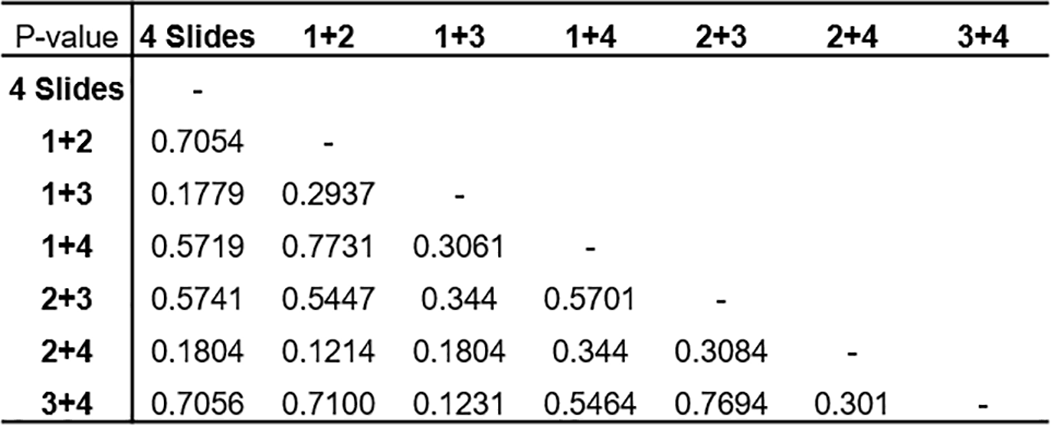

Supplement: Supplementary file 2 — Additional file 2: Table S1. CTC enumeration based on 4 slides compared to any combination of 2 slides using the HD-SCA workflow for 130 NSCLC liquid biopsy samples using the Wilcoxon matched-pairs signed rank test. [file 12967_2019_2035_MOESM2_ESM.tif]

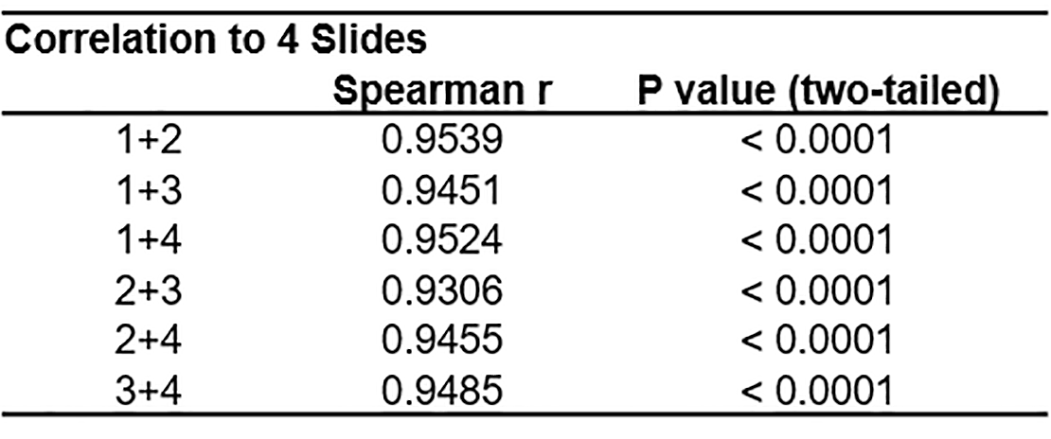

Supplement: Supplementary file 3 — Additional file 3: Table S2. Correlation between CTC enumeration based on 4 slides versus 2 slides using the HD-SCA workflow for 130 NSCLC liquid biopsy samples (Spearman’s r correlation test). [file 12967_2019_2035_MOESM3_ESM.tif]

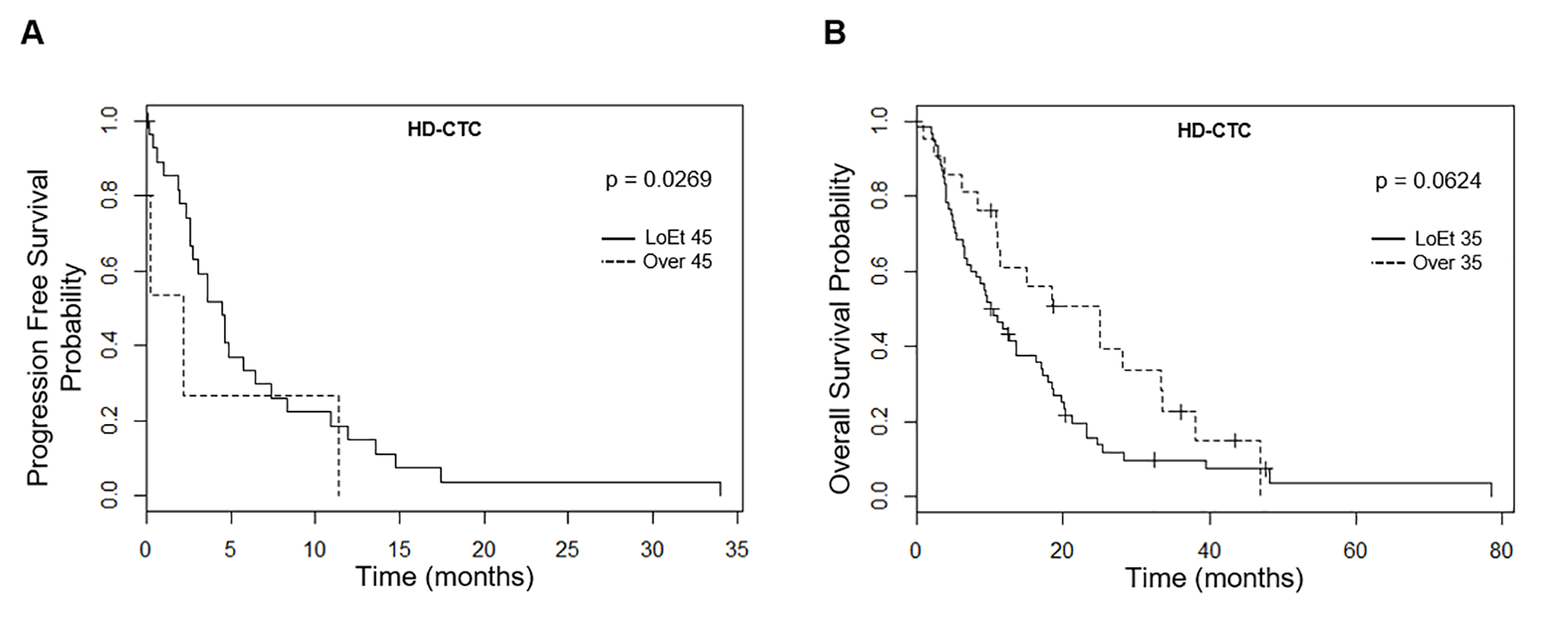

Supplement: Supplementary file 4 — Additional file 4: Figure S2. Kaplan–Meier survival analysis of PFS (A) and OS (B) of stage IV NSCLC cohort conducted similarly to Nieva et al, in which all CTC counts from multiple blood draws for each individual patient were averaged in an attempt to show a survival difference (24). This confirms the previously reported results that higher numbers of detected CTCs were associated with unfavorable prognosis in advanced NSCLC. [file 12967_2019_2035_MOESM4_ESM.tif]
